# HIV in Eswatini: Climate Change Impacts and Adaptation Strategies

**DOI:** 10.1007/s40475-024-00325-z

**Published:** 2024-07-06

**Authors:** Neliswa P. Mkhatshwa, Wisdom Mdumiseni Dlamini, Angelle Desiree LaBeaud, Anna M. Mandalakas, Kevin Lanza

**Affiliations:** 1 Wits School of Geography, Archaeology and Environmental Studies, University of the Witwatersrand, Johannesburg, South Africa; 2 Department of Geography, Environmental Science and Planning, University of Eswatini, Kwaluseni, Eswatini; 3 Department of Pediatrics (Infectious Diseases), Stanford University School of Medicine, Stanford, U.S.A.; 4 Department of Pediatrics, Baylor College of Medicine and Texas Children’s Hospital, Houston, U.S.A.; 5 Department of Environmental & Occupational Health Sciences, Department of Epidemiology, The University of Texas Health Science Center at Houston School of Public Health, Austin, U.S.A.

**Keywords:** Climate Change, HIV, Food Security, Children, Climate Adaptation, Low- and Middle-Income Countries

## Abstract

**Purpose of Review:**

This review assessed the impact of climate change on HIV transmission and HIV care of children and adults in Eswatini, and what adaptation strategies can mitigate these impacts.

**Recent Findings:**

The HIV crisis in Eswatini persists alongside the climate emergency, increasing poor health outcomes in individuals living with HIV. Although there is no clinical evidence of a direct influence of climate change on the biological effect of HIV, changing weather patterns have an effect on the livelihoods and sustenance of children, adults, and caregivers, which may consequently increase the likelihood of HIV transmission and disrupt HIV care.

**Summary:**

Drought conditions—expected to increase with climate change—coupled with existing food insecurity and poverty are the main pathways linking HIV and climate change in Eswatini. Other climate-driven concerns for HIV treatment and care in Eswatini include heat waves, wildfires, floods, and storms.

## Introduction

The Kingdom of Eswatini—a small country of approximately 1.3 million people—has the highest HIV/AIDS prevalence (31%) in adults between the ages of 15–49 years globally [1]. As of 2019, Eswatini recorded about 6,900 new HIV cases, resulting in 2,600 people dying from HIV/AIDS-related opportunistic infections [1]. HIV/AIDS has contributed to the country’s low life expectancy of 58 years [2]. Furthermore, HIV-related deaths are directly linked to high poverty rates in Eswatini [3]. In 2016, national poverty levels were 59%, which is predicted to drop to 53% in 2024 [4]. Despite the slight decrease in poverty rates, a majority of the population still survives on less than 2.15 USD a day, considered by the World Bank to be extreme poverty for a middle-income country [5].

Recent statistics show a promising trend in HIV incidence in Eswatini, from 14,000 cases in 2010 to 4,300 cases in 2023 [6]. The decrease in HIV cases is attributed to the 95–95–95 targets, an ambitious set of goals by the joint United Nations Program on HIV/AIDS (UNAIDS) that ensure that by the year 2030, 95% of people who are HIV positive should know their HIV status, 95% should be receiving treatment, and 95% receiving treatment should be virally suppressed [7]. In 2021, Eswatini, in partnership with UNAIDS and the U.S President’s Emergency Plan for AIDS Relief, was able to achieve results of 94–97–96 in adults—this means of the adult population living with HIV/AIDS, 94% knew their HIV status, 97% were receiving HIV treatment, and 96% had achieved viral load suppression [7]. The Eswatini Population-based HIV Impact Assessment specified that women achieved 95–98–96 and men achieved 92–96–97 [8].

Eswatini has implemented several interventions that have decreased the number of new HIV infections and the viral load in those with HIV. The significant progress in controlling HIV in Eswatini is partly attributed to the reduction of mother-to-child transmission by introducing new diagnostic and treatment solutions for the identification, monitoring, and treatment of infected infants [9]. Interventions include increasing HIV testing, care, and monitoring as well as improving service delivery in rural communities where there are challenges to accessing health facilities. Other programs such as voluntary male circumcision, administration of pre-exposure prophylaxis, and post-exposure prophylaxis have reduced the likelihood of HIV infection [10]. Furthermore, stigma and discrimination associated with HIV has decreased, which has encouraged more people at risk of contracting HIV to seek testing and treatment [11].

Although Eswatini has implemented HIV interventions to significantly reduce the number of new infections and achieve viral suppression, future success of these interventions is threatened by climate change, which the World Health Organization considers the greatest public health threat of the twenty-first century [12]. Studies caution that climate change in Sub-Saharan Africa may potentially influence HIV response in the future [13]. First, climate change may increase HIV transmission—the spread of the HIV virus from one positive person to a negative person through unprotected sex, exchange of body fluids, and during perinatal care [14]. Second, climate change may impact the health of HIV patients by disturbing the five stages of the HIV care continuum: [1] diagnosis of HIV, [2] linkage to HIV care, [3] receiving HIV care, [4] retained in care, and [5] achieved viral suppression [15].

The purpose of this review is to outline the impact of climate change on HIV transmission and care in Eswatini, and to recommend adaptation strategies to mitigate the impact of climate change on the HIV epidemic. We first provide an overview of Eswatini’s climate and how children have worse HIV outcomes and higher vulnerability to climate change compared to adults. Our synthesis of the literature can inform the design of interventions to moderate the effects of climate change now and in the future on HIV transmission and care in children and adults.

## Eswatini’s Climate: Baseline, Historical Trends, and Current and Future Hazards

Located in a subtropical climate, Eswatini has four physiographic zones distinguishable by elevation and subclimate: Highveld, Middleveld, Lowveld, and Lubombo Plateau [16]. Each zone aligns with an administrative region (Highveld = Hhohho, Middleveld = Manzini, Lowveld = Shiselweni, Lubombo Plateau = Lubombo). The Highveld receives the most rainfall ranging from 900–1,500 mm each summer (October–March) [17]. Middleveld and Lubombo have similar climatic conditions, with both regions receiving 700–1,000 mm of rainfall annually [17]. The Lowveld is the driest region in the country and most prone to drought, receiving less than 500 mm of annual rainfall [16]. Temperatures are high in the summer and low in the winter (April–September) when it is cold and dry. Mean average monthly temperatures range 15–23 °C across Eswatini [17], with temperatures averaging 17 °C in Highveld, 19 °C in Lubombo and Middleveld, and 22–29 °C daily in Lowveld [17].

Historical observations of Eswatini’s climate show clear trends in temperatures and precipitation. From 1901–2020, Eswatini observed a temperature increase of approximately 2 °C [16]. Days with extreme heat (i.e., temperatures exceeding 34 °C) were rarely recorded in the 1970’s yet have become more common since [16]. According to precipitation data from 1970–2010, Eswatini has also observed changes in the timing and intensity of rainfall including an increase in average dry spell length [16].

**Fig. 1:**
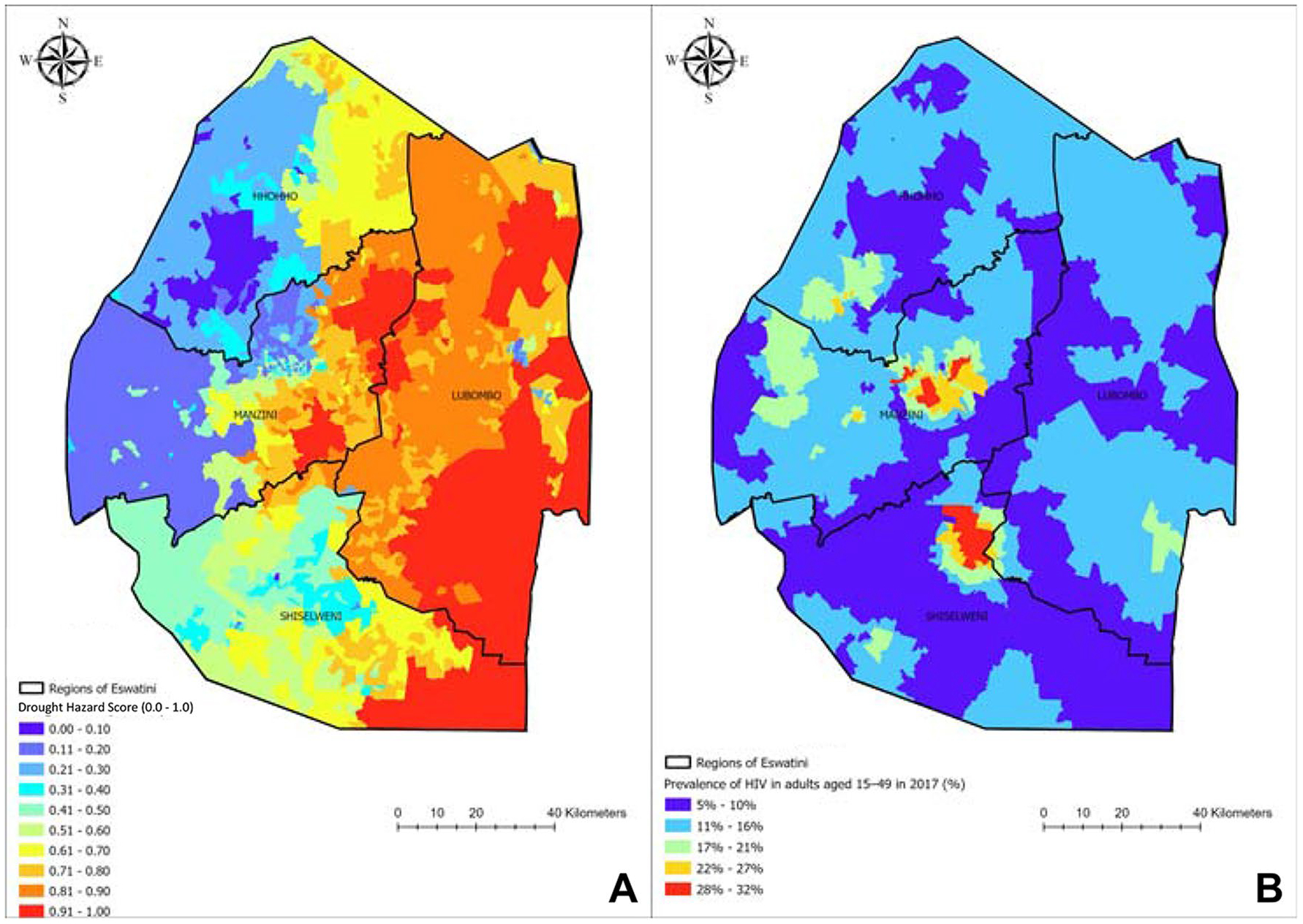
A) Drought Hazard Score (low hazard = 0.0, high hazard = 1.0) and B) prevalence of HIV (%) in adults aged 15–49 across the four regions of Eswatini in 2017

Eswatini has experienced an increase in the climate-related hazards of droughts, heat waves, wildfires, floods, and storms, with droughts and heat waves of greatest concern [18]. Droughts are an ongoing and future hazard of concern in Eswatini due to rising temperatures and declining overall precipitation levels, driving approximately 14% of the country’s population to experience drought [18]. Historically, droughts in Eswatini were recorded in 1981–1984, 1990–1992, 2001–2003, 2006–2008, 2011–2013, 2015–2016, and 2018–2019 [16]. We computed a Drought Hazard Score using data on the length of dry season and standardized precipitation evaporation index [19, 20] to reveal a west-to-east gradient from lowest-to-highest risk of drought conditions across Eswatini, with drought being a significant hazard for the majority of the Lubombo region (Fig. 1); the spatial distribution of drought is wider spread than the prevalence of HIV, the latter of which was concentrated in central Manzini and northern Shiselweni in 2017 [21]. The spatial heterogeneity between drought hazard levels and HIV cases may factor into future HIV risk across the country due to potential shifts in resources and population caused by drought conditions. Furthermore, historical data and projected weather patterns estimate Eswatini will experience an increase in droughts in the future [22].

Although limited data have been recorded on heat waves in Eswatini, climate change is expected to increase the likelihood of these extreme heat events as temperatures increase. Along with droughts and heat waves, wildfires are another climate-related hazard impacting Eswatini. In 2019, the National Disaster Management Agency declared wildfires a national emergency after approximately 36 hectares of land were lost to wildfires [23]. Floods affect approximately 1,500 people in urban areas of Eswatini annually, and the risk of flooding is expected to increase [16]. Lastly, Eswatini had a total of five storms from 2001–2021, the most intense occurring in 2005 which directly impacted about 100,000 people [24].

## Unequal impact of HIV and Climate Change on Children

With high HIV incidence and inadequate plans for mitigating climate change impacts threatening global health systems, particularly in Sub-Saharan Africa, children are more prone to poor health outcomes [25]. Approximately 2.4% of children ages 0–14 years (i.e., 10,000 children) in Eswatini were infected with HIV in 2021 [26], which was partially driven by limited access to preventative measures of transmitting HIV from mother-to-child during pregnancy, childbirth, and breastfeeding, especially for rural women and teenage mothers [27]. Children are more vulnerable to mortality from HIV because of the challenges in diagnosing HIV early, which delays treatment initiation including provision of appropriate antiretroviral therapy [27].

Children are also more vulnerable than adults to the health impacts of climate change because their bodies and immune system are still developing, which makes it difficult to recover from health hazards related to climate change [25]. Children have a breathing rate that is two-to-three times higher than adults because of their lower lung capacity [28], which means that air pollution from wildfires and other sources have a larger impact on children than adults, especially during periods of lung development in childhood. Spending more time outdoors compared to adults also puts children at a greater risk of being exposed to air and water pollution as well as other environmental stressors related to climate change [25]. Furthermore, children are disproportionately impacted by climate change because their bodies require more water intake to receive adequate hydration during their daily activities [29], placing them at greater risk of heat-related illness during periods of heat waves and water-borne diseases associated with climate change such as cholera, diarrhea, and Escherichia coli.

In addition to the effects of HIV and climate change on the physical health of children, HIV and climate change-related hazards have a greater impact on the mental health of children than adults because children may not have the coping strategies that adults do [25]. Lastly, children are oftentimes dependent on their caregivers for everyday activities and survival, so their health depends on the health and well-being of the adults in their lives. In Eswatini, women are the primary caregivers, yet have the least access to resources including land, finances, and decision-making power [30]. Survival of caregivers, particularly those in rural areas, relies heavily on the natural environment, where having limited access to land directly impacts food availability [30].

## Relationship between Climate Change, Food Insecurity, and Poverty

The impacts of climate change (i.e., extreme heat, changing precipitation patterns, and increases in natural disasters) directly affect food production and availability leading to food insecurity [31]. Climate change also impacts the accessibility of micronutrient rich crops, limiting the availability of nutritious meals [31]. Consequently, this exacerbates poverty and malnutrition as people experience limited access to food resources and nourishing meals [32].

The impact of climate change on food production limits agricultural productivity by reducing crop yields and livestock production, making it challenging to meet the demand for healthy meals in rural areas where livelihoods are sustained by subsistence farming [33]. Over 70% of the country’s population relies on subsistence farming, producing maize as the main crop [34]. Subsistence crops grown in Eswatini also include legumes, sorghum, and tubers [34]. Although oftentimes these crops are drought tolerant, increased demands for seeds and crop variants compel farmers to import from neighboring countries to increase their crop yields [34]. Imported seeds that are not indigenous to the country fail to adapt in extreme weather conditions [34]. The impact of drought on imported seeds affects subsistence farming and leads to food shortages, food insecurity, and price spikes, creating poverty [35].

Food insecurity, poverty, and decreased nutritional value in crops become chronic when subsistence farmers in rural communities lack access to sufficient and healthy meals [36]. In impoverished communities, disruption in food production and supply caused by climate change and its impacts worsen food insecurity, causing disadvantaged households and HIV patients to spend their income on costly food, further pushing them into poverty [35].

## Effects of Climate Change on HIV Transmission in Eswatini

HIV transmission in drought- and poverty-stricken communities of Eswatini may be accelerated by limited access to HIV preventative methods and treatment [37]. When residents experience extreme hunger, their priority is to purchase food, not to visit clinics and receive care for pay [38]. HIV-positive individuals who are food insecure may be more likely to transmit HIV to others because they have limited access to HIV preventative methods (e.g., condoms) and treatment (e.g., antiretroviral therapy) [37].

Food insecurity has been shown to increase the risk of young women in Eswatini engaging in transactional sex to generate income for their well-being [39], which increases the risk of contracting HIV along with other sexually transmitted diseases. Faced with poverty and food insecurity, a large percentage of young women in rural areas without tertiary education migrate to industrialized cities, where many of them find low-paying jobs in textile firms [40]. Recent government statistics show that young women present the largest population at risk of being infected with HIV in the country, in particular young women who are factory workers and those in tertiary education who engage in transactional sex in response to financial demands [41].

Poverty results in a significant percentage of young women in rural areas of Eswatini dropping out of school, a setting where they would have learned about the possible routes of HIV transmission and ways they can protect themselves from HIV [42]. The lack of knowledge and understanding of HIV and other sexually transmitted diseases has been shown to increase the chance of being infected by HIV and passing the virus to sexual partners [42]. Lack of knowledge on HIV transmission and preventative methods is also a hindrance in making informed sexual health reproductive decisions [43].

Additionally, food insecurity in Eswatini may disrupt healthcare systems that implement interventions for malnutrition deficits and other comorbidities, which can lead to HIV treatment interruptions and ultimately increase HIV transmission rates as the virus is not suppressed [44]. Overall, poor health outcomes may be observed when droughts and other climate-related hazards disrupt healthcare systems [44].

The growing threat of droughts on the agricultural sector in Eswatini may result in climate migration [45]. Migration from rural areas to inner cities where residents do not depend on subsistence agriculture increases the risk of HIV transmission, particularly for less privileged young women [46]. Finding stable and permanent employment may be challenging for recently migrated young women, which increases the chances of transactional sexual encounters that increase the risk of HIV [46]. Along with internal migrants, residents of neighboring countries may migrate to Eswatini to escape climate-related hazards—such as the frequent flooding in Mozambique—that then can influence HIV transmission [46]. Migrants entering Eswatini may lack awareness of HIV services in the country and may find that Eswatini has different HIV treatment protocols, potentially disrupting antiretroviral therapy treatment [47]. Illegal immigrants are even more vulnerable as they may not be eligible to receive adequate care until they are documented [48].

Beyond the potential impact of drought on food security and HIV transmission, weather-related hazards in Eswatini that are expected to increase with climate change—including heat waves, wildfires, floods, and storms—may serve as barriers to procuring HIV preventative methods and treatment, consequently increasing transmission risk. These hazards may make it uncomfortable or unsafe to leave their residence and seek HIV preventative methods (e.g., condoms) or travel to clinics to receive treatment that suppresses viral load (i.e., antiretroviral therapy). Individuals who typically walk, bicycle, or use public transit to get to and from clinics may be disproportionately impacted by these hazards due to their higher levels of exposure to weather elements when traveling [49].

## Effects of Climate Change on HIV Care and Treatment in Eswatini

HIV care includes effective antiretroviral therapy that protects against further health deterioration of individuals living with HIV and helps to suppress the virus [50]; however, the impacts of drought and poverty on food security threaten the responsiveness of the immune system to antiretroviral therapy because malnutrition reduces the ability of antiretroviral therapy to manage HIV, leading to higher risks of poor health outcomes [51].

Climate change disproportionately impacts the care of HIV patients experiencing comorbidities, as these other underlying illnesses can affect retention in HIV care and achievement and maintenance of viral load suppression [52]. Common comorbidities in Eswatini include tuberculosis (TB), cryptococcal meningitis, and malaria. In 2019, the dual burden of TB/HIV in Eswatini was 31%, where TB incidence was 363 cases per 100,000 people of which 66% of TB patients were already diagnosed with HIV [5]. Climate factors such as increased temperatures and changing rainfall patterns and relative humidity levels influence the spread of TB by creating an environment conducive to hosting and transmitting Mycobacterium tuberculosis, the TB bacterium [53]. Increased air pollution puts HIV patients at risk of contracting pulmonary TB, which further weakens their immune systems [54]. Furthermore, climate change impacts social factors—including food security, poverty, and displacement—that influence the spread of TB [53].

Along with TB, HIV patients in Eswatini are at higher risk of acquiring cryptococcal meningitis [55]. A national study conducted in 2014–2015 revealed that six percent of HIV patients had the cryptococcal antigen, the highest prevalence in the world [55]. Although the infection is HIV related, it can be climate sensitive, and its intensity can be linked to increasing temperatures and air pollution [56]. Extreme heat combined with airborne dust are common risk factors for meningitis, and both are exacerbated by climate change [57]. Frequency of droughts can also influence cryptococcal meningitis during dry seasons due to dust storms that pollute the air [58].

Another comorbidity of HIV in Eswatini is malaria, a disease that has a direct relationship with climate [59], which is concerning since HIV patients with malaria have poor health outcomes [60]. Increasing temperatures accelerate breeding and production rates for anopheles mosquitos and shorten the incubation period for the disease [61], increasing the risk of transmission [61]. Second, changes in rainfall patterns can create ideal environments for mosquito oviposition, which facilitates the spread of malaria [62]. Drought conditions can influence the occurrence of malaria, as prolonged periods of hot and dry weather slow the water flow of rivers, lakes, and streams and create stagnant ponds that later can become mosquito breeding grounds [63]. Extensive irrigation in drought-stricken areas can potentially increase malaria risk, especially if paired with poor canal water management [64].

Lastly, people living with HIV have a higher chance of experiencing depression and other cognitive disorders such as mood swings and anxiety, both of which carry social stigma [65]. The mental health of HIV patients can be worsened by climate change effects. Since climate-related hazards such as droughts, floods, and heat waves can disrupt HIV care, HIV patients may worry about how they will manage their HIV if there are disruptions in health systems, food availability, and water quality [66]. In addition, climate anxiety and mental health trauma from climate-related hazards [67] can exacerbate the mental health effects experienced from HIV, resulting in worse health outcomes.

## Adaptation Strategies to Mitigate the Effects of Climate Change on People Living with HIV in Eswatini

To address the ongoing and future effects of climate change on HIV transmission and care (Table 1), the Government of Eswatini can invest in drought-resistant practices such as planting of drought-tolerant crops to combat issues of food insecurity, especially in areas susceptible to drought [68]. Drought-tolerant crops play a crucial role in improving food production in arid areas by enhancing yield stability through improved soil fertility and water infiltration, reducing the chances of crop loss during periods of inadequate rainfall [69]. This ensures that children and adults have enough nutritious meals and respond well to HIV treatment and overall HIV care. Additionally, improved food security from surplus crops may limit transactional sex and sexual violence experienced by young women, which consequently may reduce the chances of HIV transmission in children and adults. In addition to planting drought-tolerant crops, the impacts of drought can be mitigated by implementing regenerative agriculture [70], an agricultural practice that encourages conservation and rehabilitation of degraded soil [71] by shifting towards farming methods that promote biodiversity and improve ecosystem functions [72]. Altogether, the effects of drought on HIV transmission and care can be moderated by implementing practices such as water-efficient landscaping by planting indigenous plants or planting crops of similar nature in the same area [73], and smart irrigation systems that improve water harvesting and soil health [74].

While the Government of Eswatini has improved the delivery of health services in rural areas [75], there needs to be enhanced efficiency of operations at HIV healthcare facilities, and provision of services beyond HIV testing and counseling. Healthcare facilities in rural communities vulnerable to climate-related hazards need to increase data monitoring and evaluation systems to ensure that HIV interventions are data driven to optimize HIV services [76]. This can be done by monitoring program effectiveness by analyzing HIV prevalence and health outcomes in rural areas [77]. This also ensures that interventions are implemented in rural areas outside of better resourced hospitals in cities.

Planting trees along travel routes and at public transit stops and stations indirectly supports HIV patients that walk, cycle, and/or use public transit to reach clinics for treatment, as trees promote general health by providing cleaner air and cooler travel environments [49]. The importance of cool travel routes is particularly important for HIV patients, whose medications and comorbidities can increase their risk of heat-related illness, and tree shade has been shown to lower heat index by approximately 6 °C compared to surrounding unshaded areas [78]. As trees also improve air quality, HIV patients with compromised immune systems may be saved from opportunistic infections such as cryptococcal meningitis that can be influenced by airborne dust [56].

During flood events, HIV patients who rely on public transit may not be able to receive essential care. Stormwater management practices called “green infrastructure” can be implemented at bus stops to reduce flood risks [79]. This includes installing rain gardens and permeable pavements to improve air quality and mitigate the intensity of the urban heat island effect, which can improve health outcomes for those with compromised immune systems [80]. When flood risk is reduced, it is safer and easier for HIV patients to access healthcare and achieve HIV care [81].

Individuals with HIV are particularly vulnerable to changing weather patterns due to the frequent need to visit healthcare centers [82]. Government officials in Eswatini could advance electronic health systems and weather early warning systems to assess and identify the risk of climate-related hazards in HIV hotspots. Such technology would enable healthcare providers to contact HIV patients to check on their ability to receive HIV care before, during, and after extreme weather events [83]. Implementing this emergency response strategy would facilitate efficient delivery of food parcels and HIV treatment to people living with HIV impacted by climate change.

Water, sanitation, and hygiene (WASH) programs are concerned with the increased access to safe drinking water [84], waste management systems, pest and vector control [64], and improving environmental conditions to minimize the risk of disease transmission [85]. Integrating WASH into HIV programs ensures that HIV transmission through unsanitary disposal of infected human waste (e.g., fecal matter, female sanitary pads) does not transmit HIV through skin contact [84]. WASH can also prevent perinatal HIV transmission in mothers living in unsanitary conditions [86].

Moreover, WASH can prevent the spread of other diseases such as cholera, diarrhea, and hepatitis A, all of which can increase chronic comorbidities in people living with HIV [87]. Overall, incorporating WASH interventions into HIV programs is crucial to HIV care because it ensures that large portions of HIV funds are not only allocated to purchasing antiretroviral drugs for HIV treatment, which can be disrupted by chronic diseases from lack of WASH [86].

In addition to integrating WASH into HIV programs, educational programs can reduce the effects of climate change on people living with HIV. This can be achieved through initiating and supporting research and data collection that investigates the specific pathways linking climate change to HIV transmission rates, treatment outcomes, and access to healthcare infrastructure [13]. We recommend engaging with communities most affected by HIV and climate change to understand experiences of affected persons [88], which can inform interventions to mitigate the impact of climate change on HIV. These interventions could then lead to development of an evidenced-based policy to integrate climate change adaptation strategies into existing HIV programs and policies in the country.

## Conclusions

We conducted a literature review that examined the relationship between climate change and HIV transmission and care in Eswatini. Through published government reports and academic journals, we revealed how Eswatini has successfully managed the spread of HIV in the country. We then analyzed historical, current, and projected climate hazards in Eswatini, and mapped HIV cases and hazard-prone areas in all four regions of the country. Next, we explained the unequal impact of HIV and climate change on children. We described that climate change is expected to increase drought conditions that compound food insecurity and poverty and may have the potential to impact HIV transmission. In Eswatini, climate change impacts may serve as a barrier to accessing HIV preventative methods and treatment, compel young women to engage in risky sexual behaviors, disrupt healthcare systems, inhibit education on sexual reproductive health, and create barriers to securing HIV preventative methods and treatment. Additionally, HIV care is impacted by droughts as it contributes to increases in malnutrition, climate migration, comorbidities, and mental health challenges. To counteract the potential impacts of climate change on HIV transmission and care in Eswatini, we recommended implementation of drought-resistant practices and regenerative agriculture, improved access to healthcare facilities, provision of green infrastructure for heat and stormwater management, enhanced electronic health systems and hazard warning systems, integration of WASH programs into HIV programs, and development and administration of educational programs on the relations between climate change and HIV transmission and care. Based on the findings of this research, we call for more evidence-based research on climate change and HIV management in Eswatini and other nations to ensure HIV health policies are inclusive of climate change adaptation strategies.

## Figures and Tables

**Table 1:** Integrating climate change adaptation strategies into HIV programs

Climate Change Adaptation Strategy	Implementation Approach
Drought-resistant practices	Plant drought-resistant and indigenous crops
	Install smart irrigation systems
	Invest in regenerative agriculture
Green infrastructure	Planting trees and other vegetation along active travel routes and transit stops/stations to promote safe and comfortable travel to healthcare facilities
	Improve stormwater management through techniques such as rain gardens and permeable pavements
Integration of electronic health systems and weather hazard warning systems	Connect electronic HIV health systems with weather warning systems to identify HIV patients in areas prone to extreme weather events
HIV-centered water, sanitation, and hygiene (WASH) programs	Ensure availability of clean and safe water, sanitation facilities, and hygiene practices in communities with high HIV prevalence and most prone to climate-related hazards
Educational programs on climate change and HIV prevention and care	Develop learning materials that incorporate information about the relationship between climate change and HIV/AIDS
